# Statistical Characterization of Strain-Controlled Low-Cycle Fatigue Behavior of Structural Steels and Aluminium Material

**DOI:** 10.3390/ma15248808

**Published:** 2022-12-09

**Authors:** Žilvinas Bazaras, Vaidas Lukoševičius

**Affiliations:** Faculty of Mechanical Engineering and Design, Kaunas University of Technology, Studentų Str. 56, 51424 Kaunas, Lithuania

**Keywords:** correlation, durability, low-cycle fatigue, regression, probability, strain-controlled loading

## Abstract

Probabilistic evaluation of the resistance to low-cycle deformation and failure of the critical components in the equipment used in the energy, engineering, metallurgy, chemical, shipbuilding, and other industries is of primary importance with the view towards their secure operation, in particular, given the high level of cyclic loading acting on the equipment during its operation. Until recently, systematic probabilistic evaluation has been generally applied to the results of statistical and fatigue investigations. Very few investigations applying this approach to the low-cycle domain. The present study aims to substantiate the use of probabilistic calculation in the low-cycle domain by systematic probabilistic evaluation of the diagrams of cyclic elastoplastic deformation and durability of the materials representing the major types of cyclic properties (hardening, softening, stabilization) and investigation of the correlation relationships between mechanical properties and cyclic deformation and failure parameters. The experimental methodology that includes the calculated design of the probabilistic fatigue curves is also developed and the curves are compared to the results of the experiment. Probabilistic values of mechanical characteristics were determined and calculated low-cycle fatigue curves corresponding to different failure probabilities, to assess them from the probabilistic perspective. A comparison of low-cycle fatigue curves has shown that the durability curves generated for some materials using analytical expressions are not accurate. According to the analysis of the relative values of experimental probabilities of low-cycle fatigue curves, the use of analytical expressions to build the curves can lead to a significant error. The results obtained allow for the revision of the load bearing capacity and life of the structural elements subjected to cyclic elastoplastic loading in view of the potential scattering of mechanical properties and resistance parameters to low-cycle deformation and failure. In addition, the results enable determination of the scatter tolerances, depending on the criticality of the part or structure.

## 1. Introduction

Individual structural elements of machinery and units have been subjected to elastoplastic, static, or cyclic deformation caused by operation under high stress conditions related to the attempt to achieve maximum performance indicators (capacity, output, speed) while maintaining minimum metal consumption [1,2]. For greater operational reliability and safety of different structures and products, probabilistic methods are used increasingly extensively to calculate their strength and durability. These methods are based primarily on the use of statistical information on the mechanical properties and durability of the material under cyclic loading [3,4,5].

The probabilistic approach to the calculation of structures and the determination of the design characteristics of materials has been developed for more than 80 years. Weibull made a considerable contribution to the development of probabilistic methods, probabilistic substantiation of the permissible stresses, and strength safety margins for the calculation of static and cyclic strength [6,7]. The ‘weakest link hypothesis’ developed by Weibull allowed the scholar to build the theory of probabilistic weak fracture of bodies under the action of stress. This helped to solve the issues of fatigue failure theory. The strain-controlled failure strength was investigated by Coffin [8], Manson [9], and Langer [10]. The study by Iida and Inoue [11] provided findings on the probability of low-cycle fatigue failure. The results of the life distribution under strain-controlled loading in low-cycle fatigue tests were investigated using the normal, log-normal, and Weibull distributions. Low-cycle fatigue durability distributions were found to be fairly consistent with the log-normal distribution and Weibull distribution rather than with the normal distribution. Scattering of the crack initiation life was found to generally exceed scattering of the durability to failure. The concept of fatigue design curves was addressed in the investigation of the strength and life reduction factors. A statistical evaluation of the fatigue characteristics of the sample tested at the same level primarily requires addressing the issue of the distribution law. Different distribution functions were proposed by Freudenthal and Gumbel [12,13] as well as other researchers in relation to this issue. Serensen, Kogayev, Shneiderovich [14,15], Stepnov [16], revised and upgraded probabilistic fatigue calculations under low-cycle fatigue strain-controlled loading, performed the analysis of random deviations of the acting stress-strain forces, and analyzed the distribution of the probability of fatigue failure according to the durability characteristics.

Several researchers contributed considerably to the calculation of probabilistic methods for the mechanical and low-cycle properties. Makhutov et al. [17] presented the results of experimental investigations and durability and life calculations of low alloy and austenitic steels with different mechanical properties. Daunys et al. [18,19] investigated the dependence of low-cycle fatigue durability on the mechanical properties of steel welds in nuclear power plants. Timofeev et al. [20] and Raslavičius et al. [21] investigated the probabilistic low-cycle fatigue life and the dependence of low-cycle durability on the mechanical properties WWER-type reactor of the nuclear power plant made of steel 15Cr2MoVA and structural steel C45. Another study [22] involved the analysis of the mechanical reliability of magnesium alloys and systematic evaluation using the statistical Weibull analysis. The results obtained are very important in terms of the safety and reliability evaluation of the magnesium alloys for lightweight structures. Zhu et al. [23] have developed a probabilistic methodology to predict low cycle fatigue life by using an energy-based damage parameter built on the Bayes theorem. Strzelecki [24] presented the characteristics of the S-N curve that employ the 2-parameter and 3-parameter Weibull distributions for fatigue limit and limited life, respectively. The parameters of the proposed model were evaluated under the maximum likelihood method. In addition, the article presented the solution to the problem of estimating the initial values of the likelihood function. Fekete [25] proposed a new model for low-cycle fatigue prediction based on strain energy to account for just the part of the microstructure of the strain energy stored in the material that causes the fatigue damage.

The above review of the work has demonstrated that, until now, statistical evaluation under low-cycle loading has only been applied to durability [26,27,28]. There are no studies dedicated to the statistical evaluation of the strain diagrams or the parameters thereof. The evaluation of the durability to the final rupture or crack initiation has generally been performed under low-cycle fatigue conditions by applying the laws of normal, log-normal, and Weibull distribution. The following could be concluded in relation to the findings of the investigation of static characteristics and low-cycle fatigue:

- The mechanical characteristics and the characteristics of resistance to low-cycle deformation and failure have not been investigated to the extent that would be enable comprehensive application of its results to the probabilistic calculations.
- In the case of low-cycle loading, there is lack of systematic data on the investigation of the laws of distribution of durability for materials with different cyclic properties (hardening, softening, stabilization), while static investigations of resistance to low-cycle deformation under strain-controlled loading are non-existent.
- It is known that the curves of low-cycle fatigue under strain-controlled loading can be built using the mechanical characteristics, for example, $\sigma_{ys},\;\sigma_{u},\;\psi$ etc. However, there are no systematic investigations that would be dedicated to determination of correlation relations between mechanical characteristics and durability.

Based on the above review of the scientific studies and the conclusions thereof, the following objectives were pursued in the study presented here: (1) Definition of the laws of distribution of the mechanical characteristics and low-cycle (deformation and durability) characteristics for the materials that contrast to each other by their cyclic properties (hardening, softening, stable) with the aim of subsequent reliable static assessment (by using the confidence intervals) of the mechanical and resistance properties of low-cycle fatigue; (2) Definition of the correlation relations between the key mechanical characteristics and parameters of low-cycle fatigue that enable building of the low-cycle fatigue curves by using the mechanical characteristics; (3) Development of the methodology for determination of the probabilistic curves of low-cycle failure on the basis of the investigation conducted.

## 2. Materials, Specimens and Methods

### 2.1. Materials

Due to the statistic nature of the fatigue failure as well as static failure, there is scattering of the material properties, which may also depend on the cyclic properties of materials and the character of failure under low-cycle loading (fatigue, quasistatic, transient failure). Therefore, the static investigations conducted in the study used the specimens produced of the materials that contrasted with each other by cyclic properties: cyclically softening low alloy steel 15Cr2MoVA, cyclically stable medium carbon steel C45, and cyclically hardening aluminium alloy D16T1. The same cast was used for production of the specimens.

The 15Cr2MoVA specimens were cut from 120 mm thick rolled stock making sure that the direction of the rolled stock corresponded to the specimen axis. Steel specimens C45 and aluminium alloy D16T1 were cut out of 50 mm diameter bar stock. The 15Cr2MoVA forging and aluminium alloy were subject to thermal processing in the following modes: hardening in oil by heating up to 1000 °C followed by two subsequent quenching sequences at 700 °C for 14 h and in 670 °C for the 70 h for steel 15Cr2MoVA and standard hardening and quenching for the aluminium alloy D16T1. The chemical composition [29,30] and mechanical characteristics of the investigated materials are presented in Table 1 and Table 2.

**Figure 1:**
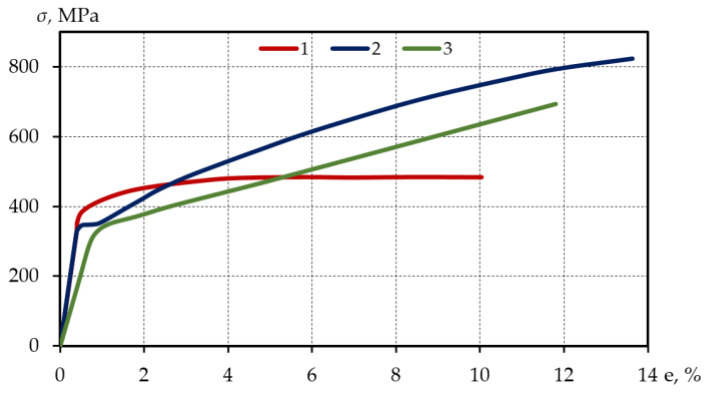
Single uniaxial tensile deformation curves: 1—aluminium alloy D16T, 2—structural steel C45, 3—alloyed steel 15Cr2MoVA.

Table 2 provides the expected value of the mechanical characteristics of the investigated materials, while Figure 1 provides the single deformation representing these characteristics, that is, the curves corresponding to the 50% probability.

Based on Table 2, steels 15Cr2MoVA and C45, especially steel 15Cr2MoVA (ψ=80%) are plastic materials. The diagram of single uniaxial tensile deformation of steel C45 is characterized by the yield plateau (up to e=0.6−0.65), while the diagrams of single uniaxial tensile deformation of steel 15Cr2MoVA and aluminum alloy D16T1 do not contain a yield plateau. The chosen materials represent key types of cyclic properties: cyclic hardening, stabilization, and failure. Therefore, the experimental and theoretical findings obtained can be applied to the assessment of a wide range of materials used in structures subject to low-cycle loading.

### 2.2. Specimens

**Figure 2:**
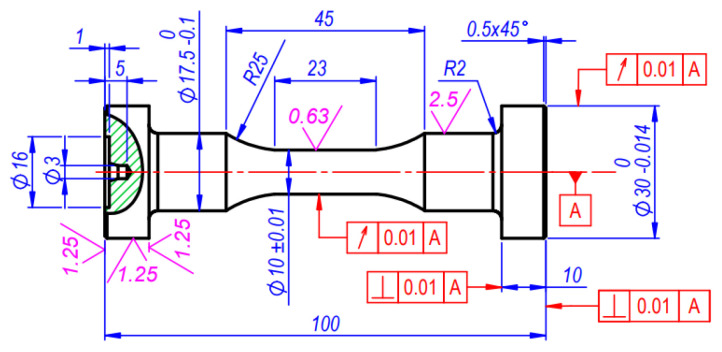
Specimens for low-cycle fatigue experiments (units in mm).

For the purpose of cyclic experiments, specimens with a cylindrical deformable part: (length—23 mm, diameter—10 mm) were selected. The cutting modes of processing were chosen to avoid traces of crushing and vibrations on the working surface. The specimens were processed with a special 60mm diameter form turning tool for all the materials investigated. A detailed drawing of the specimen is presented in Figure 2.

The specimens made of steel 15Cr2MoVA or C45 were machined with a 0.1 mm for grinding on the main part and with allowance 0.15 mm on the surface of the deformable part. The grinding of the deformable part and the base surfaces of the specimens was performed on the external grinding machine with a rounding radius of 25 mm. The minimum radial and face runout of the specimen heads in relation to the cylindrical deformable part was achieved by grinding of the surfaces on the basis of the central holes. The samples made of aluminium alloy D16T1 were processed by turning only. The final passages were performed with cutting models that provided roughness of the working and base surfaces in the detailed drawing (Figure 2).

**Figure 3:**
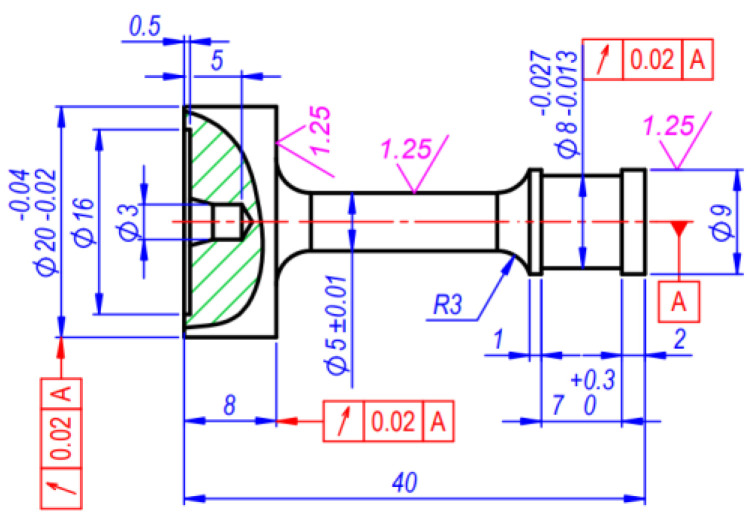
Specimens for single uniaxial tension experiments (units in mm).

After fatigue tests the fractured specimens were used as workpiece materials to produce monotonous tensile specimens with the aim of reaching the material properties nearly identical to the properties of the material subjected to cyclic loading. The detailed drawing of the specimen is presented in Figure 3. The specimens for uniaxial tension experiments have a diameter of 5 mm and 25 mm length. They were taken from the parts of cyclic test specimens (part of 17.5 mm diameter, Figure 2), that had not been subjected to plastic deformation.

To eliminate the elastoplastic bending of the specimen during production as a result of the cutting forces, the cutting depth and feed were decreasing as the diameter to be machined decreased. For the same purpose, the processing was performed using special high-speed steel cutters the geometry whereof was chosen with the view towards minimum cutting forces.

### 2.3. Methods

The experiments were carried out in the Laboratory of the Faculty of Mechanical Engineering and Design of Kaunas Technology University. The experimental equipment consisted of a 50 kN UMM-5T low-cycle tension-compression test machine (Kaunas University of Technology, Kaunas, Lithuania) and an electronic device designed to record stress-strain diagrams, cycles, and load reversal. Mechanical characteristics were measured with an error that did not exceed ±1% of the deformation scale or ±0.01% of the maximum load. GOST 25502-79 standard (Strength analysis and testing in machine building. Methods of metals mechanical testing. Methods of fatigue testing) [31] was used to perform low-cycle fatigue tests. Statistical characteristics were calculated according to the GOST 22015-76 standard (Quality of product, Regulation, and statistical quality evaluation of metal materials and products on speed-torque characteristics) [32].

**Figure 4:**
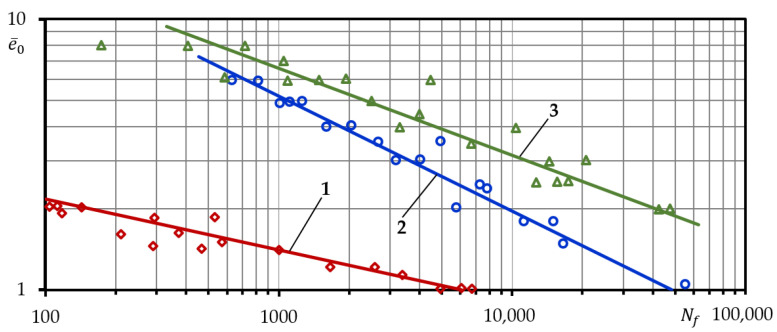
Low-cycle fatigue durability curves under strain-controlled loading: 1—aluminum alloy D16T, 2—alloyed steel 15Cr2MoVA, 3—structural steel C45.

As mentioned above, to define the patterns of statistical distribution of mechanical characteristics and parameters of low-cycle loading, three materials with contrasting cyclic properties were investigated in the study: softening steel 15Cr2MoVA, mildly softening (virtually stable) steel C45, and hardening aluminium alloy D16T1. The experiments were conducted under low-cycle strain-controlled symmetric tension and compression. Low-cycle fatigue curves under strain-controlled loading in relative coordinate system ‘total strain amplitude ${\bar{e}}_{0}$—durability to complete fracture $N_{f}$′ are presented in Figure 4.

The curves were built according to the guidelines established in the Rules and Norms in Nuclear Power Engineering (PNAE) [33]. According to the guidelines, low-cycle fatigue curves must be built after at least 15 tests on the specimens under different levels of uniformly distributed load.

The levels of loading and the number of specimens under the static investigation of each of them are presented in Table 3.

According to Table 3, static investigations under strain-controlled loading were conducted at three levels of loading. The three levels of deformation under strain-controlled loading were chosen to be uniform throughout the investigation range. Although only fatigue failure was observed under strain-controlled loading, all three levels were considered equal in relation to the type of failure. However, the number of specimens of the materials investigated at the medium level was increased in order to identify the effect of the number of specimens on the statistical characteristics of the deformation and durability diagrams.

## 3. Results

### 3.1. Statistical Investigation of the Relationship between the Mechanical Characteristics and Parameters of Resistance to Cyclic Deformation and Failure

Under strain-controlled loading, only fatigue failure is attainable due to the limitation on total deformation of the specimen due to the conditions of the experiment. Strain-controlled loading tests are generally used for the definition of failure characteristics. The most widely used Coffin—Manson equation has been applied to the strength calculation, as it defines the relationship between the size of the plastic deformation and the number of cycles to failure [8,9]:

(1) $$e_{a}N_{f}{}^{m}=C_{\Psi }$$

where m and $C_{\Psi }$ are the constants of material that, according to the Coffin data, have the following values for the majority of materials: $m=0.5{,}^{}C_{\Psi }=\frac{1}{2}\ln\frac{1}{1-\Psi }.$

Equation (1) expresses the linear relationship between plastic deformation and the number of cycles to failure in coordinate system $\mathrm{lg}e_{p}–\mathrm{lg}N_{f}$. Plastic deformation changes in the process of strain-controlled loading and is constant only for cyclically stable materials. Hence, in Equation (1), the authors recommend using value $e_{a},$ that corresponds to 50% of the loading cycles to failure, i.e., when the process of width stabilization of the elastoplastic hysteresis loop starts. Manson, when testing Equation (1), found that, for 29 materials with contrasting cyclic properties, constant m=0.6. Manson expressed the relationship between total elastoplastic deformation and the number of cycles to failure as a single dependency. The amplitude of total deformation $e_{a}$ was calculated as a sum of amplitudes of plastic $e_{p}$ and elastic strain $e_{y}$, i.e.,

(2) $$e_{a}=e_{p}+e_{y}=\frac{1}{2}{\left(\ln\frac{100}{100-\Psi }\right)}^{0.6}N_{f}{}^{-0.6}+1.75\frac{\sigma_{u}}{E}N_{f}{}^{-0.12}$$

Based on the equation by Langer [10], to determine the failure amplitudes of deformation $e_{a}$ and conditional stresses $\sigma_{a}^{*}$ under strain-controlled symmetric loading, the following dependencies were proposed:

(3) $$e_{a}=\frac{1}{4e_{t}}\frac{1}{N_{f}^{m}}\ln\frac{100}{100-\Psi }+0.4\frac{\sigma_{u}}{Ee_{t}}$$

(4) $$\sigma_{a}^{*}=\frac{1}{4}\frac{E}{N_{f}^{m}}\ln\frac{100}{100-\Psi }+0.4\sigma_{u}$$

where m—constant equal to 0.5 under $\sigma_{u}\leq 687$ (MPa).

The above dependencies are often used by designers for the calculation of heavily loaded parts and structures under low-cycle deformation conditions.

The prepared version of PNAE proposes calculating the elastoplastic deformation by using the dependency:

(5) $$e_{a}=\frac{0.5\ln\frac{100}{100-\Psi }}{{\left(4N_{f}\right)}^{0.5}}+\frac{\sigma_{u}}{E{\left(4N_{f}\right)}^{0.05}}$$

According to the investigations by Daunys [34], the following could be written down for the majority of materials:

(6) $$e_{a}N_{f}{}^{\alpha_{1p}}=C_{1p}$$

In contrast to Coffin–Manson Equation (1), in this case $\alpha_{1p}<m$ and $C_{1p}<C_{\Psi }$. Constants $\alpha_{1p}$ and $C_{1p}$ can preliminarily be defined according to the mechanical properties of the material:

(7) $$\alpha_{1p}=0.17+0.55\Psi \frac{\sigma_{ys}}{\sigma_{u}},\;C_{1p}=0.75\alpha_{1p}\ln\frac{100}{100-\Psi }$$

Similarly, the same study [34] attempted to link the parameters of the generalized curve of cyclic deformation $A_{1},\;A_{2},\;\propto ,\;c,\;{\bar{S}}_{T}$ to the mechanical properties of material. The following was obtained:

(8) $$A_{1}=0.3+0.6\Psi \frac{\sigma_{ys}}{\sigma_{u}}$$

(9) $$A_{2}=0.32{\left(\frac{\sigma_{u}}{\sigma_{ys}}\right)}^{-7}+A_{1}$$

(10) $$\propto =0.9+2.6\Psi \frac{\sigma_{ys}}{\sigma_{u}}$$

(11) $$c=0.13-0.21\Psi \frac{\sigma_{ys}}{\sigma_{u}}$$

(12) $${\bar{S}}_{T}=2-0.83\Psi \frac{\sigma_{ys}}{\sigma_{u}}$$

The analysis of the dependencies proposed by different authors for the calculation of structures and elements under elastoplastic deformation conditions has shown that durability $N_{c}$ and $N_{f}$ and parameters of the generalized diagram of cyclic deformation $A_{1},\;A_{2},\;\propto ,\;c,\;{\bar{S}}_{T}$ are often linked by the dependencies that are used for the calculation of mechanical characteristics.

However, the scientific literature reviewed did not investigate the level of correlation between these parameters. Therefore, the present study includes investigation of the correlation relations between $N_{c}$, $N_{f}$ and $\sigma_{ys},\;\sigma_{u},\;\sigma_{f},\;\psi ,\psi_{u},\;\psi \frac{\sigma_{ys}}{\sigma_{u}}\;.$ The results were processed according to the known methods of mathematical statistics [35].

The coefficient of correlation between two correlating values was determined as follows:

(13) $$r=\frac{m_{xy}}{\sigma_{x}\sigma_{y}}$$

where the second central mixed moment was determined according to the following dependency:

(14) $$m_{xy}=\frac{1}{n}\left[\sum_{1}^{n}x_{i}y_{i}-\frac{\sum_{1}^{n}x_{i}\sum_{1}^{n}y_{i}}{n}\right]$$

while root mean square deviations of the investigated correlating quantities were determined according to the following dependencies:

(15) $$\sigma_{x}=\sqrt{\frac{1}{n}\sum_{1}^{n}\left(x_{i}^{2}-{\bar{x}}^{2}\right)},\;\sigma_{y}=\sqrt{\frac{1}{n}\sum_{1}^{n}\left(y_{i}^{2}-{\bar{y}}^{2}\right)}$$

The mean arithmetic values of the investigated correlating quantities were determined according to the following dependencies:

(16) $$\bar{x}=\frac{1}{n}\sum_{1}^{n}x_{i},\;\bar{y}=\frac{1}{n}\sum_{1}^{n}y_{i}$$

The values of coefficients of regression b between the correlating quantities were determined on the basis of the following expressions:

(17) bxy=rσxσy, byx=rσyσx

In the course of calculation of the value of the coefficient of correlation r, it can approximately be assumed that the estimate thereof has been distributed normally. Therefore, the confidence interval of the valid values r is:

(18) $$r\pm t_{\gamma }\frac{1-r^{2}}{\sqrt{n}}$$

while in case of γ=0.90 and $t_{\gamma }=1.65$ [36]:

(19) $$r\pm 1.65\frac{1-r^{2}}{\sqrt{n}}$$

The linear regression equation can be brought into the following form:

(20) (x−x¯)=bxy(y−y¯)

**Figure 5:**
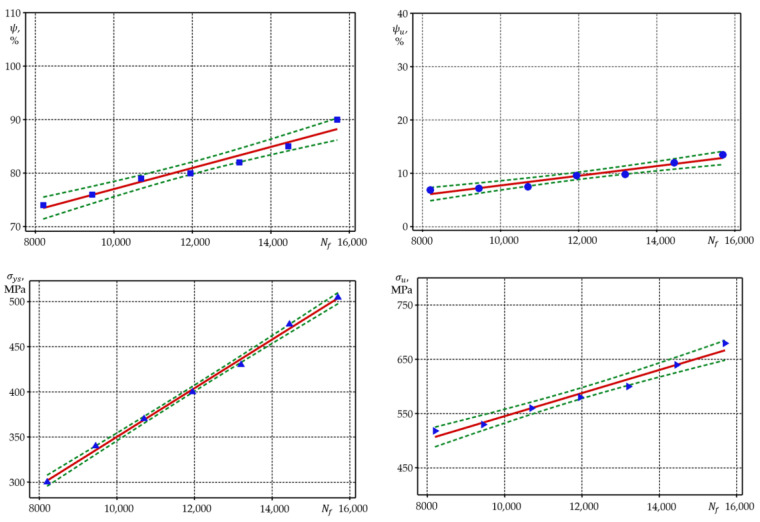

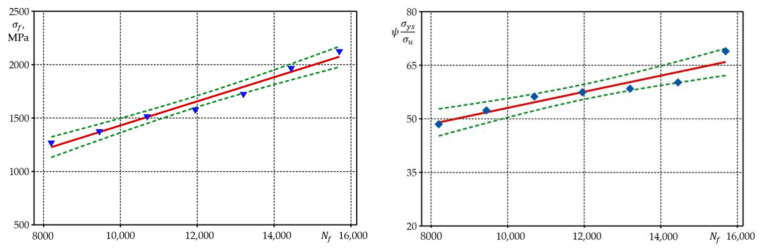
Regression lines under strain-controlled loading for steel 15Cr2MoVA (${\bar{e}}_{0}=1.8)$. Dots represent the experimental values, straight lines—the theoretical calculated dependencies, while dashed lines represent confidence intervals.

The analysis of the results of calculation of correlation coefficients has suggested that, under strain-controlled loading, durability $N_{c}$ and $N_{f}$ correlates very well (almost linearly) with mechanical characteristics $\sigma_{ys},\;\sigma_{u},\;\sigma_{f},\;\psi ,\psi_{u},\;\psi \frac{\sigma_{ys}}{\sigma_{u}}\;$ for all the investigated materials with contrasting cyclic properties. For steel 15Cr2MoVA, there is almost linear correlation with durability $N_{c}$ and $N_{f}\;$ yield strength $\sigma_{ys}$ and true fracture strength $\sigma_{f}$ (Figure 5).

According to Figure 5, there is a close correlation between the durability and mechanical characteristics of the 15Cr2MoVA steel. Similar results were obtained for the regression coefficients of the resistance characteristics to cyclic deformation in relation to the mechanical characteristics.

For steel C45, at the loading level ${\bar{e}}_{0}=2.5$ the correlation is better with durability $N_{c}$ and $N_{f}$—reduction of area ψ, while at the level ${\bar{e}}_{0}=4.0$, the correlation is better with true fracture strength $\sigma_{f}$ and yield strength $\sigma_{ys}$. For the aluminium alloy D16T1, yield strength $\sigma_{u}$ correlates directly to durability. Interestingly, for steels 15Cr2MoVA and C45, multiplier $\psi \frac{\sigma_{ys}}{\sigma_{u}}$ insignificantly increases the coefficient of correlation between $N_{c}$,$N_{f}$ and $\psi \frac{\sigma_{ys}}{\sigma_{u}}.$ For aluminium alloy D16T1, introduction of multiplier $\psi \frac{\sigma_{ys}}{\sigma_{u}}$ leads to certain reduction of the coefficient of correlation for the levels of strain-controlled loading ${\bar{e}}_{0}=1.0;1.5$, while for level ${\bar{e}}_{0}=2.0,\;$ the multiplier increases the coefficient of correlation, same as in the case of steels 15Cr2MoVA and C45.

### 3.2. Statistical Assessment of the Low-Cycle Fatigue Curves under Strain-Controlled Loading

Until present, the low-cycle fatigue curves under strain-controlled loading have been built using Equations (1)–(6) or similar equations by applying the mechanical characteristics that correspond to a probability of 50%. Therefore, the curves calculated here correspond to the same failure probability. There are no systematic investigations into the building of calculated probabilistic low-cycle fatigue curves in the scientific literature.

In this study, probabilistic values of mechanical characteristics were determined. This enabled the authors to build the calculated low-cycle fatigue curves corresponding to different failure probabilities and to assess them from the probabilistic perspective.

**Figure 6:**
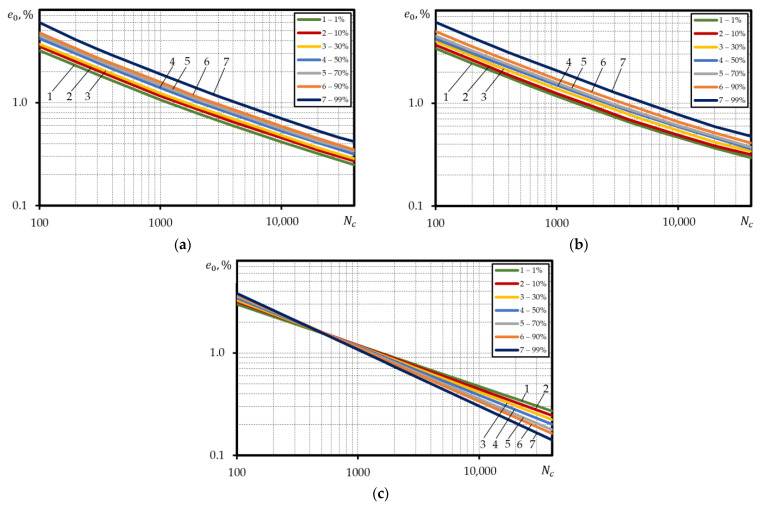
Calculated probabilistic low-cycle fatigue curves under strain-controlled loading for steel 15Cr2MoVA, built using the absolute coordinates, according to: (**a**)—Equation (3), (**b**)—Equation (5), (**c**)—Equation (6).

Table A1, Table A2 and Table A3 contain the values $\sigma_{ys},\;\sigma_{u},\;\psi ,\;e_{pr},$ corresponding to 1%, 10%, 30%, 50%, 70%, 90%, 99% probabilities for all the materials investigated. The low-cycle fatigue curves under strain-controlled loading corresponding to 1%, 10%, 30%, 50%, 70%, 90%, 99% failure probabilities for the investigated steel 15Cr2MoVA were built using Equations (3), (5) and (6) and the values of equal probability of mechanical characteristics (Figure 6a–c). The calculated curves were built using the absolute coordinates $\mathrm{lg}e_{0}-\mathrm{lg}N_{c}$.

Based on Figure 6 the results of calculations using Equations (3) and (5), are little different from each other in terms of both the slope and occupied scatter band of the probabilistic low-cycle fatigue curves. The ratio between durability values for 99% and 1% curves is little dependent on the deformation level. The average ratio for the curves calculated using Equation (3) was about 3.2, for curves calculated using Equation (5)—about 3.3. Calculation of the probabilistic low-cycle fatigue curves using Equation (6) for steel 15Cr2MoVA generates a non-satisfactory result as the curves cross each other in case of $N_{f}=200-400$ cycle durability or are positioned in reverse order in case of durabilities $N_{f}>400$, i.e., the durability is the lowest in case of 99% failure probability and the highest in case of 1% failure probability. This is associated with dependency of $\alpha_{1p}$ and $C_{1p}$ on the mechanical characteristics of the material. As follows from Equations (7) and (8), the probabilistic value of $\alpha_{1p}$ largely depends on ψ, as the probabilistic values of ratio $\sigma_{ys}/\sigma_{u}$ differ very little (Table A1). Constant $C_{1p}$ that depends on both $\alpha_{1p}$ and ψ changes within the range from 0.42 for probability curve 1% to 0.96 for probability curve 99%. Constant $\alpha_{1p}$ ranges from 0.41 (failure probability curve 1%) to 0.56 (failure probability curve 99%). The specified changes $\alpha_{1p}$ and $C_{1p}$ lead to the positioning of the probabilistic curves as depicted in Figure 6c.

**Figure 7:**
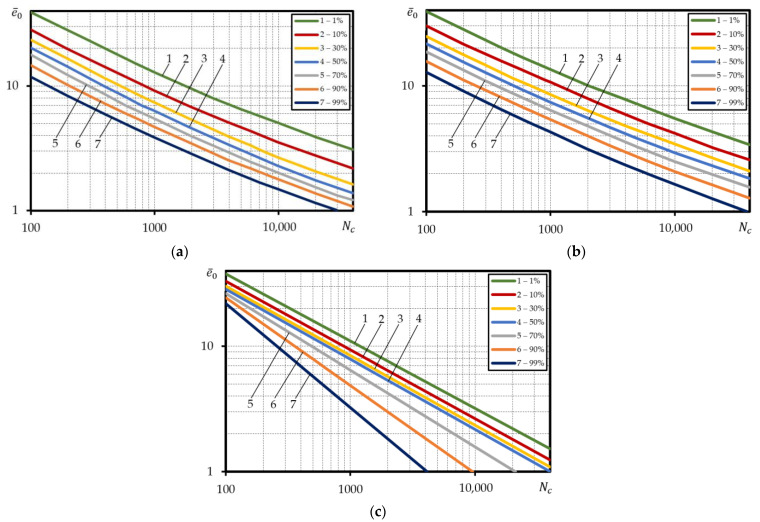
Calculated probabilistic low-cycle fatigue curves under strain-controlled loading for steel 15Cr2MoVA, built in the relative coordinates, according to: (**a**)—Equation (3), (**b**)—Equation (5), (**c**)—Equation (6).

The dependencies built in the relative coordinates are used for calculation of the low-cycle fatigue of parts and structural elements. Hence, the probabilistic curves of low-cycle fatigue under strain-controlled loading for steel 15Cr2MoVA were also built in the relative coordinates using Equations (3), (5) and (6). The amplitude strains of curve 1% were divided by probabilities $e_{pr}$—1%, strains of curve 10%—by probabilities $e_{pr}$—10%, etc. Hence, the designed calculated probabilistic curves of low-cycle fatigue in the coordinates $\mathrm{lg}{\bar{e}}_{0}-\mathrm{lg}N_{c}$ are presented in Figure 7a–c.

Figure 7 suggests that the application of the relative coordinates for the design of probabilistic curves of low-cycle fatigue generates an implausible picture for steel 15Cr2MoVA. The implausibility of the mutual position of the calculated probabilistic curves lies in that curve 99% is characterized by the lowest durability, while curve 1%—the highest durability. The ‘reverse’ layout of the probabilistic curves of low-cycle durability is related to very vast scatter $e_{pr}$ compared to the scatter of other mechanical characteristics, for example, ψ, that largely determine the durability. As suggested by Table A1, for steel 15Cr2MoVA, the ratio of values $e_{pr}$ for probability 99% to 1% probability is 7.6, while the ratio of values ψ for probability 99% to 1% probability—1.4.

The sharp contrast in the values of the scatter between $e_{pr}$ on one side and $\sigma_{ys}$, $\sigma_{u},\psi$ on the other side is likely to be due to higher sensitivity $e_{pr}$ to thermal processing, hardening during mechanical processing, accuracy of the experiment, and other factors, in comparison to other mechanical characteristics. The conducted analysis of the calculated probabilistic curves for steel 15Cr2MoVA suggests that the probabilistic values of strain $e_{pr},$ cannot be used for the design of probabilistic curves of low-cycle fatigue in the relative coordinates as they distort the true layout of the curves.

**Figure 8:**
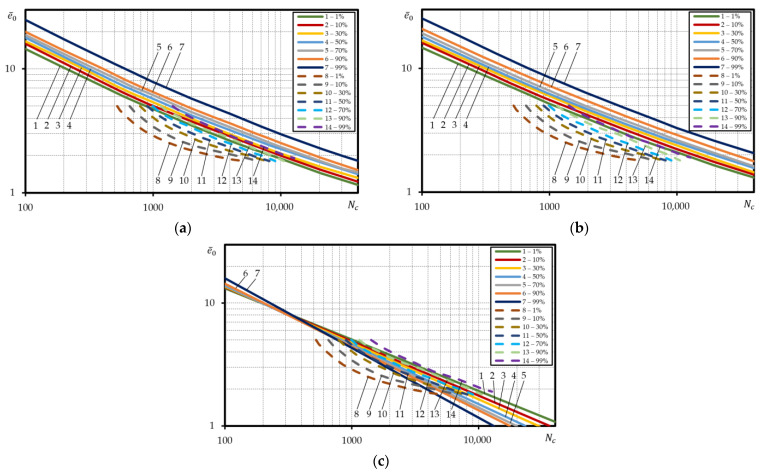
Comparison of the calculated probabilistic low-cycle fatigue curves under strain-controlled loading with the experimental ones for steel 115Cr2MoVA; straight lines—calculated, dashed lines—experiment; according to: (**a**)—Equation (3), (**b**)—Equation (5), (**c**)—Equation (6).

To define more truthful layout of the calculated probabilistic curves of low-cycle fatigue under strain-controlled loading in the relative coordinates for steel 15Cr2MoVA, the percentage curve strain was divided by the mean arithmetic value of $e_{pr}$ (Figure 8a–c).

Here, as suggested by Figure 8a–c there is little difference in the layout of the probabilistic curves in case of the absolute coordinates (Figure 6). For the calculated curves built according to Equations (3) and (5), the ratio of durability for curve 99% and curve 1% was little dependent on the strain. The average ratio for the curves built according to Equation (3) was about 2.9, and for the curves built according to Equation (5)—3.2.

To validate the calculation for steel 15Cr2MoVA, the experimental curves of equal probability were compared to the calculated curves. Figure 8a,b suggests that the slope angle and the occupied scatter band of the experimental curves of equal probability is little different from those of the calculated curves designed according to Equations (3) and (5). Nonetheless, the experimental curves were located lower than the calculated curves. For example, at low durability, experimental curve 99% corresponded to calculated curve 30% (Equation (3)), while experimental curve 50% corresponded to calculated curve 1% (Figure 8a). Correspondence between the experimental curves and calculated curves according to Equation (5) was less accurate at high durability. In this case, experimental curve 99% was calculated above than calculated area 1% for $N_{c}>3000$ cycle durability. In case of durabilities $N_{c}<3000$, experimental curve 99% corresponded to calculated curve 1% (Figure 8b). Figure 8c suggests that the calculated probabilistic curves designed according to Equation (6) completely fall within the zone of the experimental curves. Nonetheless, the comparison renders the ‘reverse’ layout of the calculated probabilistic curves impossible in the area of $N_{c}>200-400$ cycle durabilities. The reasons have already been covered above.

Similar analysis was conducted with the calculated and experimental probabilistic low-cycle fatigue curves under strain-controlled loading for steel C45. Equations (3), (5) and (6) were used to calculate the curves of equal probability by applying the probabilistic values of mechanical characteristics (Table A2). The obtained results were built using the absolute coordinates $\mathrm{lg}e_{0}-\mathrm{lg}N_{c}$. For steel C45, same as for steel 15Cr2MoVA, the probabilistic calculated curves obtained according to Equations (3) and (5) were positioned in a similar way in terms of both the slope angle and the occupied scatter band. For probabilistic curves calculated according to Equation (6), increase accompanied by higher failure probability was observed, same as in the case of steel 15Cr2MoVA. For steel C45, however, the larger range of variation of the probabilistic value and smaller range of variation of probabilistic value $C_{1p},$ than for steel 15Cr2MoVA lead to regular layout of the probabilistic curves, i.e., curve 1% provides the lowest durability, while curve 99%—the highest durability.

**Figure 9:**
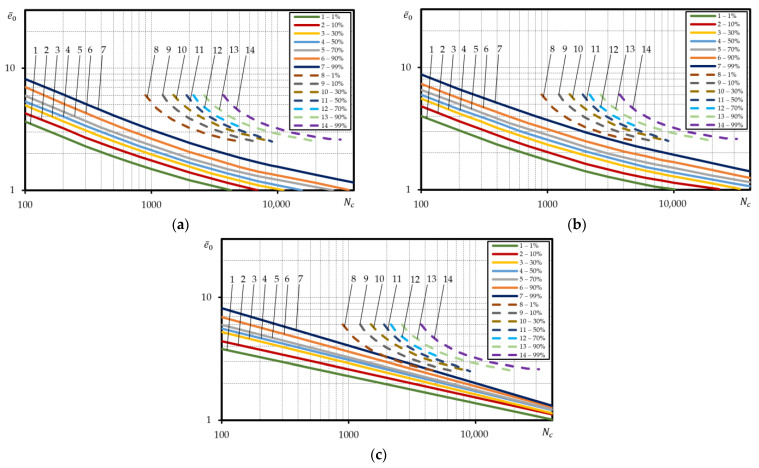
Comparison of the calculated probabilistic low-cycle fatigue curves under strain-controlled loading with the experimental ones for steel C45; straight lines—calculated, dashed lines—experiment; according to: (**a**)—Equation (3), (**b**)—Equation (5), (**c**)—Equation (6).

The investigation of the durability scatter band for steel C45 has demonstrated that the ratio of durability for probabilistic curves 99% and 1% depends on the strain level. As suggested by the analysis performed, for curves designed according to Equations (3), (5) and (6), the ratio of durability of curves 99% and 1% at strain amplitude $e_{0}=0.9\%$ was 7.3; 8.2; 10.6 respectively, while at $e_{0}=0.4\%$ — 10; 18.7; 3.7. For steel C45, same as for steel 15Cr2MoVA, the use of probabilistic value $e_{pr}$ for design of probabilistic calculated curves in the relative coordinates $\mathrm{lg}{\bar{e}}_{0}-\mathrm{lg}N_{c}$ distorts their true layout in terms of all the dependencies applied Equations (3), (5) and (6), i.e., the curves are positioned in the reverse order. At strain amplitude ${\bar{e}}_{0}=4$, the ratio of durability of probabilistic curves 1% and 99% was respective 2.8; 3.5; 8.3, and at ${\bar{e}}_{0}=2$ — 4.0; 6.7; 20.7. To obtain a valid layout of the calculated probabilistic curves in the relative coordinates for steel 15Cr2MoVA and steel C45, strains $e_{0}$ were divided by mean arithmetic value $e_{pr}$. Hence, the obtained probabilistic curves are depicted in Figure 9a–c.

The calculated probabilistic curves are close to the curves in the absolute coordinates by the layout character and slope angle. For these curves, the durability ratio of the curves 99% and 1% depend on the strain level, the same as for the curves in the absolute coordinates, i.e., at strain amplitude ${\bar{e}}_{0}=4$, the ratio of durability according to Equations (3), (5) and (6) is 7.1; 7.6; 10.3, and at ${\bar{e}}_{0}=2$ — 8.4; 11.7; 5.3.

At the same time, the same figures also include the experimental low-cycle fatigue curves under strain-controlled loading for steel C45 (Figure 9a–c). Comparison of the experimental probabilistic curves with the calculated one has shown that the calculated probabilistic curves for steel C45 (Figure 9a–c) are located below the 1% experimental probabilistic curve. Of all the dependencies applied to the calculation of probabilistic low-cycle fatigue curves under strain-controlled loading, the calculated (Equation (6)) for steel C45 was the closest to reality as demonstrated by the investigation.

The calculations of the probabilistic low-cycle fatigue curves under strain-controlled loading for aluminum alloy D16T1 (Table A3) were performed according to Equation (6), as Equations (3) and (5) were designed for low-alloy steels used for energy purpose. The same methodology was used for the design of the calculated curves as for the steels 15Cr2MoVA and C45, that is, the curves were designed in the absolute coordinates and in the relative coordinates by using probabilistic and mean arithmetic values $e_{pr}$.

**Figure 10:**
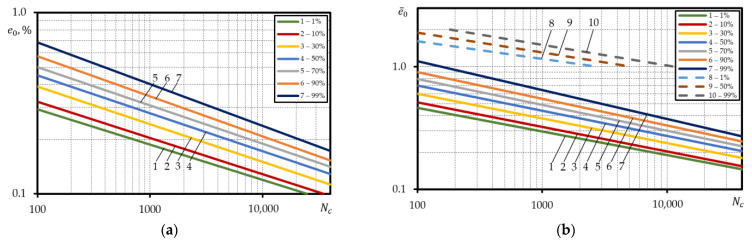
Probabilistic low-cycle fatigue curves under strain-controlled loading for aluminium alloy D16T1 built using: the absolute coordinates (**a**); the relative coordinates (**b**); straight lines—calculated, dashed lines—experiment.

Investigating the durability scatter band for the D16T1 aluminium alloy has demonstrated that the ratio for probabilistic curves 99% and 1% depends on the strain level (Figure 10).

The conducted analysis has shown that the ratio of durability at strain amplitude ${\bar{e}}_{0}=0.3$ was 37, and at ${\bar{e}}_{0}=0.18\%\;$— 24. At the same time, in the case of the calculated probabilistic curves, an increase in the slope angle with the increase in the failure probability has been observed. This is directly affected by the scatter of reduction of area ψ, same as for steels 15Cr2MoVA and C45. Application of relative strain ${\bar{e}}_{0},$ determined according to the probabilistic values of strain $e_{pr}$ to the calculations leads to narrowing of the durability scatter band. In this case, the ratio of durability of the probabilistic curves 99% and 1% at strain amplitude ${\bar{e}}_{0}=4\;$ was 3.3, and at ${\bar{e}}_{0}=3-2.7$. The slope angles of the calculated probabilistic curves in the relative coordinates were reducing in comparison to the same curves in the absolute coordinates. However, the curve angle of the slope increased with increasing failure probability.

Same as for steels 15Cr2MoVA and C45, to calculate the strains ${\bar{e}}_{0},$ of aluminium alloy D16T1, mean arithmetic value $e_{pr}$ was used. In this case, the ratio of durability of probabilistic curves 99% and 1% was close to the results of the ratio of durabilities of the probabilistic curves designed in the absolute coordinates, i.e., at ${\bar{e}}_{0}=4$, the ratio was 3.3, and at ${\bar{e}}_{0}=3-2.7$ (Figure 10b). Figure 10b also depicts the comparison of the calculated probabilistic curves with the experimental ones for aluminium alloy D16T1. As suggested by Figure 10b the correspondence of the experiment results with the calculated results is non-satisfactory, as the calculated curves are fully reflected in the elastic area.

**Figure 11:**
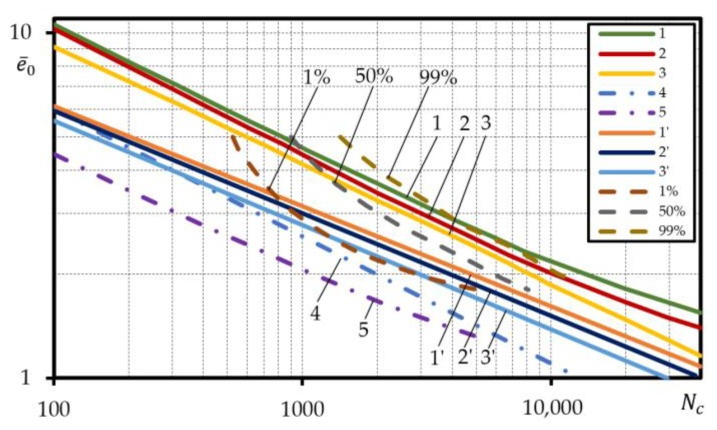
Comparison of the probabilistic low-cycle fatigue curves calculated under strain-controlled loading with the experimental ones for steel 15Cr2MoVA; straight lines—calculated, dashed lines– experiment; normalized characteristic according to: 1—Equation (3), 2—Equation (5), 3—Equation (6); reference characteristics, according to: 1′—Equation (3), 2′—Equation (5), 3′—Equation (6); calculated, according to: 4—Equation (3) $n_{e}=2,$ 5—Equation (3) $n_{N}=10$.

**Figure 12:**
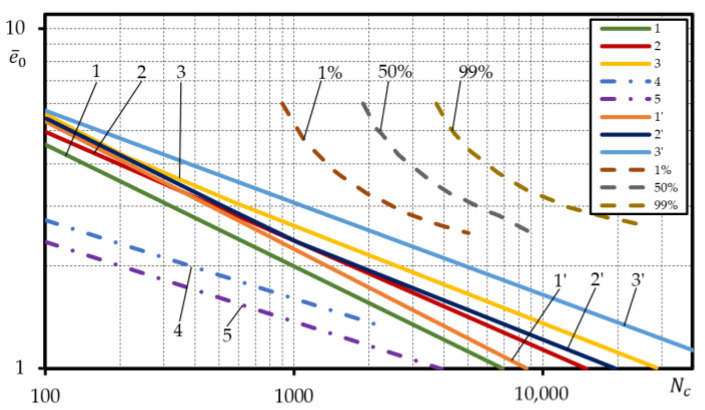
Comparison of the probabilistic low-cycle fatigue curves calculated under strain-controlled loading with the experimental ones for steel C45; straight lines—calculated, dashed lines– experiment; normalized characteristic according to: 1—Equation (3), 2—Equation (5), 3—Equation (6); reference characteristics, according to: 1′—Equation (3), 2′—Equation (5), 3′—Equation (6); calculated, according to: 4—Equation (3) if $n_{e}=2,$ 5—Equation (3) if $n_{N}=10$.

Figure 11 and Figure 12 for steels 15Cr2MoVA and C45 compare the experimental low-cycle fatigue curves under strain-controlled loading of failure probability 1%, 50%, 99% with the calculated curves designed according to Equations (3), (5) and (6) by using the normalized mechanical characteristics determined according to Equation (21) and mechanical characteristics taken from reference documents [37], and the low-cycle fatigue curves defined using the safety factor $n_{N}=10$ for cycles and $n_{e}=2$ for strain as used in the field of mechanical engineering.

(21) $$K=\frac{x_{\max}}{x_{\min}}$$

Table A4 provides the values of ratios K of the highest and lowest mechanical characteristics $\sigma_{pr},\sigma_{ys},\;\sigma_{u},\;\sigma_{f},\psi ,\;\psi_{u}$.

The design of the last curves employed the low-cycle fatigue curves designed according to the reference mechanical characteristics and the dependencies providing the most accurate description of experimental durability, i.e., Equation (3) for steel 15Cr2MoVA and Equation (6) for steel C45.

As suggested in Figure 11, for steel 15Cr2MoVA, the low-cycle fatigue curves determined according to Equations (3), (5) and (6) using normalized mechanical characteristics are above the experimental curve of probability of failure 99%. The same curves designed by using the reference mechanical characteristics are located in the durability band between curves 1% and 50%. This was predictable, as the normalized mechanical characteristics of the steel 15Cr2MoVA are close to the experimental mechanical characteristics with a probability of 12% to 25%, while the reference mechanical characteristics are located in the probability band of the experimental characteristics of 0.0003 to 74%. Due to the ‘high’ layout of the calculated low-cycle fatigue curves compared to the experimental ones, the curves designed using safety factors $n_{e}=2$ and $n_{N}=10$ are also positioned fairly high. The low-cycle fatigue curve designed using $n_{e}=2$ virtually corresponds to the experimental curve of failure probability 1%, while the curve designed using $n_{N}=10$ is located below.

Another situation is presented in Figure 12 that shows the listed low-cycle fatigue curves for steel C45. In this case, the calculated curves designed using both the normalized and the reference mechanical characteristics are located considerably lower than the experimental ones. However, this is the consequence of poor correspondence of the calculated curves designed according to Equation (6) with the experimental curves for the steel C45 (Figure 9c).

### 3.3. Case Study Objective

To Determine the Probabilistic Values of Cumulative Durability Damage (at the Crack Initiation Phase) for the Zone of Sleeve Connection to the Vessel Body under Hydraulic Forging at Temperature 20°C. The Nominal Strain Range for the Outer Surface of the Sleeve Connection under Hydraulic Forging: $\varepsilon_{1n}=0,\;\varepsilon_{2n}=0.54\%,\;\varepsilon_{3n}=-0.97\%.$ Concentration Factor (Theoretical) of Elastic Stress $\alpha_{\sigma }=3$, Strain-Controlled Loading Mode, Vessel Material—Grade 15Cr2MoVA Steel.

Mechanical and cyclic characteristics determined during the course of the study (Table A1 and Table A5) were used in the calculations.

All calculations were carried out for the failure probability of 1%, 10%, 30%, 50%, 70%, 90%, 99%. The case study presents the calculation for probability 1%. For other probabilities, the calculated values are presented in A6. The calculation was carried out as follows.

Hardening rate $m_{0}$ (Table A5) was used to design the tensile stress-strain diagrams $\bar{\sigma }-\bar{e}$. Their linear approximation resulted in following relative linear hardening modules:

(22) $$G_{T}=\frac{\bar{\sigma }-1}{\bar{e}-1},$$

$$G_{T}=\frac{2.06-1}{40-1}=0.0272$$

Parameter $\chi_{1}$ characterizing the sensitivity to cycle asymmetry was determined according to the following dependency:

(23) $$\chi_{1}=2.7\Psi \frac{\sigma_{ys}}{\sigma_{u}}-1,$$

$$\chi_{1}=2.7\cdot 0.74\frac{300}{500}-1=0.1988$$

(24) $$\chi_{2}=0.23(\frac{\sigma_{u}}{\sigma_{ys}}-1)+\chi_{1},\;\chi_{2}=0.23(\frac{500}{300}-1)+0.1988=0.3521$$

The coefficient of the cycle asymmetry intensity of the nominal stresses was assumed to be $r_{\sigma_{n}}=-1.05$, and the range of intensity of nominal stresses for the first semi-cycle of loading was determined according to the following dependency:

(25) $${\bar{S}}_{inK}=\frac{2G_{T}\left[{\bar{\varepsilon }}_{inK}{\bar{S}}_{T}-B\cdot A_{1;2}\cdot F(k)\right]}{{\bar{S}}_{T}\left[2G_{T}+p_{1;2}\cdot A_{1;2}\cdot F(k)\right]},$$

$${\bar{S}}_{inK}=\frac{2\cdot 0.0272\left[9.37\cdot 1.16-37.19\cdot 0.23\cdot 1\right]}{1.15\left[2\cdot 0.0272+1.0048\cdot 0.23\cdot 1\right]}$$

where

(26) $$p_{1;2}=1+\chi_{1;2}\frac{1+r_{\sigma }{}_{{}_{n}}}{1-r_{\sigma }{}_{{}_{n}}},$$

$$p_{1}=1+0.1988\frac{1+1.05}{1-1.05}=1.0048,$$

(27) $$B=1-\frac{1}{G_{T}}-\frac{{\bar{S}}_{T}}{2},$$

$$B=1-\frac{1}{0.0272}-\frac{1.15}{2}=-37.19$$

Hence, to calculate the Poisson’s ratio in normal section $\mu_{n}$ for the first semi-cycle of loading, it was necessary to use the value of the relative linear hardening module $g_{n1}$, and in the first approximation after replacement of the sign of main strains with the opposite sign, the following was assumed:

(28) $${\bar{\varepsilon }}_{in1}={\bar{\varepsilon }}_{1}{}_{n1},\;{\bar{\varepsilon }}_{1n1}=\frac{\varepsilon_{in1}}{e_{pr}{\bar{S}}_{T}},$$

$${\bar{\varepsilon }}_{in1}=\frac{0.97}{0.09\cdot 1.15}=9.37$$

Linear hardening module:

(29) $$g_{n1}=\frac{{\bar{S}}_{in1}-1}{\varepsilon_{in1}-1},$$

$$g_{n1}=\frac{3.0231-1}{9.37-1}=0.2417$$

and, accordingly, the Poisson’s ratio:

(30) $$\mu_{n1}=0.5-0.2\frac{1-g_{n1}({\bar{\varepsilon }}_{in1}-1)}{{\bar{\varepsilon }}_{in1}},$$

$$\mu_{n1}=0.5-0.2\frac{1-0.2417(9.37-1)}{9.37}=0.4355$$

Intensity of nominal strains of the first approximation were determined according to dependency [34]:

(31) $$\varepsilon_{in1}=\frac{\sqrt{2}}{2(1+\mu_{n}{}_{1})}\sqrt{{\left(e_{1}-e_{2}\right)}^{2}+{\left(e_{2}-e_{3}\right)}^{2}+{\left(e_{3}-e_{1}\right)}^{2}},$$

$$\varepsilon_{in1}=\frac{\sqrt{2}}{2(1+0.4355)}\sqrt{{\left(0.97-0.54\right)}^{2}+{\left(0.54\right)}^{2}+{\left(-0.97\right)}^{2}}=0.5764$$

In relative units:

$$\varepsilon_{in1}=\frac{0.5864}{0.09\cdot 1.15}=5.6657$$

The obtained value and dependencies were used to repeat the calculation and determine final values ${\bar{S}}_{in1,\;}g_{n1},\mu_{n1}$ and ${\bar{\varepsilon }}_{in1\;}$(Table A5). If symmetrical cycle of strain intensity in the nominal section was accepted for the task considered, then:

(32) $${\bar{e}}_{in}=\frac{{\bar{\varepsilon }}_{in1}{\bar{S}}_{T}}{1-r_{en}}$$

The coefficient of asymmetry of the intensity cycle of nominal strain was assumed as $r_{en}=-1$, then:

$${\bar{e}}_{in}=\frac{4.8279\cdot 1.15}{1+1}=2.7761$$

The range of main stress in the first semi-cycle of loading in the nominal section was determined according to the following dependencies:

(33) $$S_{1n1}=\frac{\varepsilon_{1n1}+\mu_{n1}\varepsilon_{2n1}}{1-\mu_{n1}^{2}}{E^{\prime }}_{1},S_{2n1}=\frac{\varepsilon_{2n1}+\mu_{n1}\varepsilon_{1n1}}{1-\mu_{n1}^{2}}{E^{\prime }}_{1}$$

$${E^{\prime }}_{1}=\frac{S_{in1}}{\varepsilon_{in1}}=\frac{2.3744\cdot 205}{4.8279\cdot 0.0009}=112,023\mathrm{MPa}$$

$$S_{1n1}=\frac{0.0097+0.4151\cdot 0.0054}{1-0.4151^{2}}\cdot 112,023=1616\mathrm{MPa},$$

$$S_{2n1}=\frac{0.054+0.4151\cdot 0.0097}{1-0.4151^{2}}\cdot 112,023=1276\mathrm{MPa}$$

In accordance with the ranges of main stress available, the strain-controlled stiffness coefficients were determined according to the dependency:

(34) $$D_{e}=\frac{\sqrt{{\left(S_{1n1}-S_{2n1}\right)}^{2}+{\left(S_{2n1}-S_{3n1}\right)}^{2}+{\left(S_{3n1}-S_{1n1}\right)}^{2}}}{\sqrt{2}(\sigma_{1}+\sigma_{2}+\sigma_{3})},\;$$

$$D_{e}=\frac{\sqrt{{\left(1616-1276\right)}^{2}+{\left(1276\right)}^{2}+{\left(-1616\right)}^{2}}}{\sqrt{2}(1616+1276)}=0.5103$$

By using ${\bar{e}}_{in},\alpha_{B},r_{en},\chi_{1},\chi_{2},\;G_{T}$ (Table A5) and $A_{1},A_{2},{\bar{S}}_{T},\beta$ as well as the Matlab programme of calculations designed according to the dependencies in papers [32], the data characterizing the stress-strain state in the zone of maximum concentration were determined (Table A6). In the durability calculation, the same as in relation to the durability calculation norms [33], the safety factor for maximum deformations $n_{e}=2.$ was accepted. Then, the cumulative quasi-static damage [34]:

(35) $$d_{k}=\frac{2({\bar{e}}_{i}-{\bar{\sigma }}_{i})}{{\bar{e}}_{B}D_{e}},\;$$

Based on study [38], ${\bar{e}}_{B}=m_{0}$ was assumed:

$${\bar{e}}_{B}=\frac{m_{0}}{e_{pr}}=\frac{0.15}{0.0009}=166.6$$

then

$$d_{k}=\frac{2\cdot (15.9-1.3405)}{166.6\cdot 0.5103}=0.3424,\;\mathrm{and}\;d_{y}=1-d_{k}=0.6576$$

The number of semi-cycles $k_{0}$, within which fatigue damage $d_{y}=0.6576$ would be cumulated was determined according to the following dependencies:

(36) $$d_{y}=\frac{\sum_{1}^{k_{0}}\frac{{\bar{\delta }}_{ik}}{D_{e}}{\left(\frac{{\bar{\delta }}_{ik}}{D_{e}}+{\bar{S}}_{ik}{\bar{S}}_{T}\right)}^{\alpha_{3}}}{C_{2}C_{3}^{\alpha_{3}}}$$

Durability $k_{0}$ calculations were conducted by using the following two techniques:

(a) in the calculations, probabilistic values of characteristics $A_{1},A_{2},\chi_{1},\chi_{2},{\bar{S}}_{T},G_{T},\beta ,D_{e},C_{2},C_{3}$ and $\alpha_{3}$ were used:
  $$0.7259=\frac{\sum_{1}^{k_{0}}\frac{2{\bar{\delta }}_{ik}}{0.5103}{\left(\frac{2{\bar{\delta }}_{ik}}{0.5103}+{\bar{S}}_{ik}\cdot 1.28\right)}^{0.98}}{500\cdot 286^{0.98}},\;k=12.5\;\mathrm{or}\;N=6$$
(b) in the calculations, the values of probability 50% of characteristics $A_{1},A_{2},\chi_{1},\chi_{2},{\bar{S}}_{T},G_{T},\beta ,D_{e}$ and probabilistic values of parameters $C_{2},C_{3}$ and $\alpha_{3}$ were used:
  $$0.6576=\frac{\sum_{1}^{k_{0}}\frac{{\bar{\delta }}_{ik}}{0.5103}{\left(\frac{2{\bar{\delta }}_{ik}}{0.5103}+{\bar{S}}_{ik}\cdot 1.15\right)}^{0.98}}{500\cdot 286^{0.98}},k=59.5\;\mathrm{or}\;N=30$$

The results of probabilistic calculations are presented in Table 4.

The total number of start-stop operations of the system for the vessel considered expected during the lifetime is equal to 25 [33]. Hence, fatigue cracks may appear in the sleeve-to-body connection during the lifetime of the vessel considered at failure probability 30% according to calculation (a) and at failure probability lower than 1% according to calculation (b).

## 4. Conclusions

The methodology of conduction of an integrated experiment in a probabilistic setting to investigate the parameters of resistance to cyclic deformation and failure for the materials representing major types of cyclic properties (hardening, softening, stabilization).

The investigations presented in the paper point to the correlation relationship between mechanical characteristics and durability as well as the cyclic deformation that is close to the linear correlation. This supports the correctness and physical rationale of the mathematical dependencies proposed by different authors for describing the low-cycle deformation and failure process under strain-controlled loading. The regression coefficients allow for the calculation of preliminary cyclic characteristics and durability using the available mechanical characteristics.

The analysis of mutual layout of the calculated and experimental probabilistic low-cycle fatigue curves conducted in the study once again has demonstrated that the design of low-cycle fatigue curves according to the both analytical dependencies for specific materials may lead to considerable errors. Hence, in calculations of critical structures, it is necessary to have at least an experimental curve of failure probability 50% to conduct a reliable assessment of the strength and durability of the structure considered.

Investigations have pointed at the presence of a stable correlation relationship between mechanical characteristics and durability as well as the parameters of cyclic deformation parameters. This supports the physical rationale of the phenomenological dependencies proposed by different authors for the description of the low-cycle deformation and failure process under strain-controlled loading by using mechanical characteristics.

The above investigation has suggested that probabilistic curves should not be built in the relative coordinates using the probabilistic $e_{pr}$, if scatter $e_{pr}$ is considerably larger than scatter $\sigma_{ys}$, $\sigma_{u}$ and ψ. Equations (3), (5) and (6) are not universal, since, for steel 15Cr2MoVA, the best correspondence is provided by Equations (3) and (5), while for steel C45 Equation (6).

The statistical investigations conducted in the paper have shown that that phenomenological dependencies used at present for the description of low-cycle fatigue curves on the basis of mechanical characteristics are not universal for the materials with contrasting cyclic properties, and a reliable assessment of the durability of structure requires the probabilistic experimental curve 50%. For example, the calculated low-cycle fatigue curves under strain-controlled loading defined using the safety factor [31] $\left(n_{e}=2,\;N_{n}=10\right)$ and the calculated curves that provide more accurate description of the experimental data provide the following strength safety margins in comparison to the experimental curves:$\;n_{e}=0.45,\;N_{n}=4.10$ (for steel 15Cr2MoVA) and $n_{e}=4.85,\;N_{n}=100$ (for steel C45).

## Figures and Tables

**Table 1:** Chemical composition of the materials.

Material	C	Si	Mn	Cr	Ni	Mo	V	S	P	Mg	Cu	Al
						%						
15Cr2MoVA (GOST 5632-2014)	0.18	0.27	0.43	2.7	0.17	0.67	0.30	0.019	0.013	-	-	-
C45 (GOST 1050-2013)	0.46	0.28	0.63	0.18	0.22	-	-	0.038	0.035	-	-	-
D16T1 (GOST 4784-97)	-	-	0.70	-	-	-	-	-	-	1.6	4.5	9.32

**Table 2:** Mechanical properties of the materials.

Material	$e_{pr}\;$	$\sigma_{pr}\;$	$\sigma_{ys}$	$\sigma_{u}$	$\sigma_{f}\;$	ψ
	%		MPa			%
15Cr2MoVA (GOST 5632-2014)	0.200	280	400	580	1560	80
C45 (GOST 1050-2013)	0.260	340	340	800	1150	39
D16T1 (GOST 4784-97)	0.600	290	350	680	780	14

**Table 3:** Specification of low-cycle strain-controlled loading (${\bar{e}}_{0}=\mathrm{constant})$.

Material	$\mathrm{Loading}\;\mathrm{Level},\;{\bar{e}}_{0}$	Number of Specimens, Pcs.
15Cr2MoVA	1.8	40
	3.0	80
	5.0	40
C45	2.5	50
	4.0	120
	6.0	50
D16T1	1.0	20
	1.5	80
	2.0	20

**Table 4:** Results of probabilistic calculation of life in the concentration zone.

Parameter	Probability, %						
	1	10	30	50	70	90	99
$k_{0}$	12.5	21.5	52.0	86.0	201.0	287.0	389.0
$k_{0}$	59.5	66.5	78.5	86.0	98.5	123.0	171.0

## Nomenclature

Latin symbols	
$A_{1},\;A_{2}$	constants of the low–cycle fatigue curve under strain-controlled loading describing the first and second semi-cycle form respectively;
B	parameter $\left(B=1-\frac{1}{G_{T}}-\frac{{\bar{S}}_{T}}{2}\right)$;
b,bxy, byx	regression coefficients;
$C_{2},\;C_{3},\propto ,\;c$	constants of the low–cycle fatigue curve under strain-controlled loading;
$C_{1p},\;C_{\psi }$	constants of the Coffin–Manson equation;
$D_{e}$	strain-controlled stiffness coefficient;
$d_{k}$	cumulated quasi-static damage;
$d_{y}$	cumulated fatigue damage;
E	modulus of elasticity (MPa);
$E_{1}^{\prime }$	secant modulus for the first semi-cycle diagram (MPa);
$E_{T\;},E_{Tk}$	linear hardening modules for single and cyclic (k-th semi-cycle) loading respectively (GPa);
e	strain intensity under linear strain-controlled state (%);
$e_{1},e_{2},e_{3}$	principal strain (%);
$e_{i}$	strain intensity (%);
$e_{in}$	nominal strain intensity (%);
$\bar{e},\;{\bar{e}}_{i},\;{\bar{e}}_{in}$	respective deformations in relative units $\left({\bar{e}}_{i}=e_{i}/e_{pr},\;{\bar{e}}_{in}=e_{in}/e_{pr}\right)$, (%);
$e_{0}$	intensity of maximum strain at the initial loading under the linear strain-controlled state (%);
${\bar{e}}_{0}$	intensity of maximum strain at the initial loading normalized to proportional limit strain;
$e_{a}$	total strain amplitude (%);
$e_{y}$	amplitude of elastic strain (%);
$e_{p}$	amplitude of plastic strain (%);
$e_{pr}$	proportional limit strain (%);
$e_{t}$	material plasticity indicator determined by assessment of the variation in the cross-section area of the standard cylindrical specimen subjected to tension;
i=1…n	specimen ranks in the rank order;
$F\left(k\right)$	function of k semi-cycles [36];
$G_{T},g_{ni}$	relative linear hardening modules for single and cyclic loading diagrams respectively $\left(G_{T}=E_{T}/E,\;g_{k}=E_{Tk}/E\right)$, (GPa);
k	number of loading semi-cycle;
$k_{0}$	number of semi-cycles of the loading cycles to fatigue crack initiation;
K	coefficient *K* values of relative measures of the key mechanical properties;
m	constant of the Coffin–Manson equation;
$m_{0}$	hardening parameter for the tensile diagram under its stepped approximation;
$m_{xy}$	second central mixed moment;
$N_{0}$	number of loading cycles to fatigue crack initiation;
$N_{c}$	durability (number of load cycles) until crack initiation;
$N_{f}$	durability (number of load cycles) till the cracks propagated to complete rupture;
n	number of of measurements;
$n_{e}$	safety factor of strength by maximum deformations;
$n_{N}$	safety factor of strength by the number of cycles;
$p_{1;2}$	parameter $\left(p_{1;2}=1+\chi_{1;2}\frac{1+r_{\sigma n}}{1-r_{\sigma n}}\right)$;
r	coefficient of correlation;
$r_{en}$	coefficient of asymmetry of the intensity cycle of nominal;
$r_{\sigma_{n}}$	coefficient of asymmetry of the intensity cycle of nominal stress;
S	cyclic stress intensity under linear strain-controlled state, calculated from the start of unloading (MPa);
$S_{i}$	cyclic stress intensity (MPa);
$S_{in}$	nominal cyclic stress intensity (MPa);
$S_{k,}S_{ik,}S_{ink}$	respective cyclic strain for the k-th semi-cycle of loading (MPa);
${\bar{S}}_{k,}{\bar{S}}_{ik,}{\bar{S}}_{ink}$	respective cyclic strains in relative units $\left({\bar{S}}_{k}=S_{k}/S_{T},\;{\bar{S}}_{ik}=S_{ik}/S_{T},\;{\bar{S}}_{ik}=S_{ink}/S_{T}\right)$, (MPa);
$S_{T}$	cyclic limit of proportionality calculated from the start of unloading (MPa);
${\bar{S}}_{T}$	cyclic stress normalized respectively to proportional limit stress $\left({\bar{S}}_{T}=S_{T}/\sigma_{pr}\right)$_,_ (MPa);
$t_{\gamma }$	Student’s t-distribution;
$\bar{x},\bar{y}$	statistical mean;
$x_{i},y_{i}$	independent variables;
$x_{max}$	maximum values of the material mechanical properties in the bins (statistical intervals);
$x_{min}$	minimum values of the material mechanical properties in the bins (statistical intervals);
Greek symbols	
α,β	constants of the low–cycle fatigue curve under strain-controlled loading characterizing materials hardening or softening;
$\alpha_{1p}$	constant of the Coffin–Manson equation;
$\alpha_{\sigma }$	theoretical coefficient of concentration of elastic stresses;
γ	reliability of normal distribution;
δ	intensity of cyclic plastic deformations under linear strain-controlled states or width of the elastoplastic hysteresis loop;
$\varepsilon_{1n},{\;\varepsilon }_{2n},{\;\varepsilon }_{3n}$	range of main nominal strains (%);
ε	cyclic elastoplastic strain intensity under linear strain-controlled state, calculated from the start of unloading (%);
$\varepsilon_{i}$	cyclic elastoplastic strain intensity (%);
$\varepsilon_{in}$	nominal cyclic elastoplastic strain intensity (%);
$\varepsilon_{k,}\varepsilon_{ik,}\varepsilon_{ink}$	respective cyclic strains for the *k*-th semi-cycle of loading (%);
${\bar{\varepsilon }}_{k,}{\bar{\varepsilon }}_{ik,}{\bar{\varepsilon }}_{ink}$	respective cyclic strain in relative units $\left({\bar{\varepsilon }}_{k}=\varepsilon_{k}/\varepsilon_{T},\;\;{\bar{\varepsilon }}_{ik}=\varepsilon_{ik}/\varepsilon_{T},\;{\bar{\varepsilon }}_{ink}=\varepsilon_{ink}/\varepsilon_{T}\right)$;
$\varepsilon_{T}$	strain corresponding to $S_{T}$ (%);
${\bar{\varepsilon }}_{T}$	strain corresponding to ${\bar{S}}_{T}$ (%);
$\mu_{ni}$	Poisson’s ratio for elastoplastic deformation;
$\sigma_{x},\sigma_{y}$	root mean square deviation;
$\sigma_{a}^{*}$	conditional elastic stresses $\left(\sigma_{a}^{*}=e_{0}Ee_{t}\right)$, (MPa);
$\sigma_{pr}$	proportional limit stress (MPa);
$\sigma_{ys}$	elastic limit or yield strength, the stress at which 0.2% plastic strain occurs (MPa);
$\sigma_{u}$	ultimate tensile stress (MPa);
$\sigma_{f}$	fracture strength (MPa);
$\bar{\sigma }$	normalized to proportional limit stress (MPa), $\bar{\sigma }=\sigma /\sigma_{pr}$;
$\chi_{1},\;\chi_{2}$	parameter characterizing the sensitivity to cycle asymmetry;
ψ	cross-sectional narrowing (%);
$\psi_{u}$	continuous cross-sectional narrowing (%).

## Appendix A

**Table A1:** Probabilistic values of mechanical characteristics for steel 15Cr2MoVA.

Characteristic	Probability, %						
	1	10	30	50	70	90	99
$\sigma_{ys}$, MPa	300	340	370	400	430	475	535
$\sigma_{u}$, MPa	500	530	560	580	600	640	680
ψ, %	74	76	79	80	82	85	90
$e_{pr}$, %	0.090	0.130	0.170	0.200	0.245	0.320	0.475

**Table A2:** Probabilistic values of mechanical characteristics for steel C45.

Characteristic	Probability, %						
	1	10	30	50	70	90	99
$\sigma_{ys}$, MPa	220	265	300	340	360	420	500
$\sigma_{u}$, MPa	620	700	750	800	850	900	1020
ψ, %	28	32	37	39	42	47	54
$e_{pr}$, %	0.140	0.180	0.225	0.260	0.300	0.360	0.480

**Table A3:** Probabilistic values of mechanical characteristics for aluminium alloy D16T1.

Characteristic	Probability, %						
	1	10	30	50	70	90	99
$\sigma_{ys}$, MPa	260	300	320	350	370	405	460
$\sigma_{u}$, MPa	580	620	650	680	700	750	800
ψ, %	9.5	11.3	12.8	14.0	15.5	17.5	21.0
$e_{pr}$, %	0.46	0.52	0.56	0.60	0.64	0.70	0.78

**Table A4:** Coefficient *K* values of relative measures of the key mechanical properties.

Material	*σ_pr_*	*σ_ys_*	*σ_u_*	*σ_f_*	*ψ*	*ψ_u_*
		MPA			%	
15Cr2MoVa	2.06	1.88	1.52	1.78	1.24	4.19
C45	2.41	2.41	1.57	1.62	1.71	3.09
D16T1	1.72	1.66	1.45	1.43	2.24	1.38

**Table A5:** Probabilistic values of parameters of cyclic deformation resistance for steel 15Cr2MoVA.

Parameter	Probability, %						
	1	10	30	50	70	90	99
$A_{1}$	0.23	0.87	1.32	1.60	1.94	2.40	3.04
$A_{1}$	0.30	0.94	1.34	1.64	2.00	2.43	3.06
${\bar{S}}_{T}$	1.15	1.20	1.25	1.28	1.30	1.35	1.40
$m_{0}$	0.15	0.17	0.19	0.21	0.23	0.26	0.30

**Table A6:** Calculation data in the concentration zone for failure probabilities 1%, 10%, 30%,50%, 70%, 90%, 99%.

Parameter	Probability, %						
	1	10	30	50	70	90	99
$G_{T}$	0.0272	0.0328	0.0400	0.0451	0.0497	0.0605	0.0754
$\chi_{1}$	0.1988	0.3164	0.4093	0.4896	0.5867	0.7033	0.9118
$\chi_{2}$	0.3521	0.4449	0.5274	0.5931	0.6776	0.7832	0.9741
$p_{1}$	1.0048	1.0077	1.0099	1.0119	1.0143	1.0171	1.0222
B	−37.19	−30.08	−24.62	−21.81	−20.06	−16.20	−12.96
${\bar{\varepsilon }}_{1n1}$	9.37	6.16	4.56	3.79	3.04	2.26	1.46
${\bar{S}}_{1n1}$	3.0231	1.9699	1.7305	1.6435	1.5871	1.4779	1.3771
$g_{n1}$	0.2417	0.1879	0.2049	0.2300	2.2870	0.3786	0.8223
$\varepsilon_{in1}$	0.4355	0.4360	0.4242	0.4133	0.3958	0.3693	0.3111
${\bar{e}}_{in}$	2.7761	0.23073	1.9606	1.5412	1.2836	1.0122	0.7181
β	0.00004	0.00016	0.00042	0.00085	0.0018	0.0048	0.0190
$E_{1}^{\prime }$	112,023	92,705	87,001	90,992	94,974	98,169	105,772
$S_{1n1}$	1616	1315	1199	1226	1236	1220	1227
$S_{2n1}$	1276	1031	929	939	931	895	858
$D_{e}$	0.5103	0.5109	0.5119	0.5130	0.5146	0.5174	0.5230
$\alpha_{\sigma }$	3	3	3	3	3	3	3
$r_{en}$	−1	−1	−1	−1	−1	−1	−1
$d_{k}$	0.3424	0.3443	0.2976	0.2741	0.2551	0.1232	0.2197
$d_{y}$	0.6576	0.6557	0.7024	0.7259	0.7449	0.7802	0.8768
